# PET staging of amyloidosis using striatum

**DOI:** 10.1016/j.jalz.2018.04.011

**Published:** 2018-05-21

**Authors:** Bernard J. Hanseeuw, Rebecca A. Betensky, Elizabeth C. Mormino, Aaron P. Schultz, Jorge Sepulcre, John A. Becker, Heidi I. L. Jacobs, Rachel F. Buckley, Molly R. LaPoint, Patrizia Vannini, Nancy J. Donovan, Jasmeer P. Chhatwal, Gad A. Marshall, Kathryn V. Papp, Rebecca E. Amariglio, Dorene M. Rentz, Reisa A. Sperling, Keith A. Johnson, the Alzheimer’s Disease Neuroimaging Initiative and the Harvard Aging Brain Study

**Affiliations:** a Department of Radiology, Massachusetts General Hospital, Harvard Medical School, and the Martinos Center for Biomedical Imaging, Charlestown, MA, USA; b Department of Neurology, Cliniques Universitaires Saint-Luc, Institute of Neurosciences, Université Catholique de Louvain, Brussels, Belgium; c Department of Neurology, Massachusetts General Hospital, Harvard Medical School, Boston, MA, USA; d Department of Biostatistics, Harvard T.H. Chan School of Public Health, Boston, MA, USA; e Faculty of Health, Medicine and Life Sciences, School for Mental Health and Neuroscience, Alzheimer Centre Limburg, Maastricht University, Maastricht, The Netherlands; f Center for Alzheimer Research and Treatment, Department of Neurology, Brigham and Women’s Hospital, Harvard Medical School, Boston, MA, USA

**Keywords:** Amyloid PET imaging, Structural MRI, Striatum, Cortex, Cognitive aging, MCI, Alzheimer’s disease, Classification, Staging

## Abstract

**Introduction:**

Amyloid positron emission tomography (PET) data are commonly expressed as binary measures of cortical deposition. However, not all individuals with high cortical amyloid will experience rapid cognitive decline. Motivated by postmortem data, we evaluated a three-stage PET classification: low cortical; high cortical, low striatal; and high cortical, high striatal amyloid; hypothesizing this model could better reflect Alzheimer’s dementia progression than a model based only on cortical measures.

**Methods:**

We classified PET data from 1433 participants (646 normal, 574 mild cognitive impairment, and 213 AD), explored the successive involvement of cortex and striatum using 3-year follow-up PET data, and evaluated the associations between PET stages, hippocampal volumes, and cognition.

**Results:**

Follow-up data indicated that PET detects amyloid first in cortex and then in striatum. Our three-category staging including striatum better predicted hippocampal volumes and subsequent cognition than a three-category staging including only cortical amyloid.

**Discussion:**

PET can evaluate amyloid expansion from cortex to subcortex. Using striatal signal as a marker of advanced amyloidosis may increase predictive power in Alzheimer’s dementia research.

## 1. Introduction

Brain amyloid β (Aβ) deposition, one of the defining pathologies of Alzheimer’s disease, is now detectable *in vivo* with high specificity using PET, as confirmed at autopsy [1–3]. It is well established that elevated PET measures of brain Aβ increase risk for subsequent cognitive decline in both cognitively impaired [4] and normal populations [5–8], and Aβ measures have been widely adopted as part of eligibility criteria for anti-Aβ therapeutic trials. However, the subsequent decline of clinically normal (CN) individuals with elevated Aβ occurs slowly over several years [9], and alternative PET measures that could predict decline over a shorter interval could potentially improve the efficiency of prevention trials.

We explored an alternative PET measure seeking to stage Aβ pathology *in vivo* based on the established Thal-Phase postmortem ordinal system for regional extent of Aβ pathology [10–12]. We evaluated the predictive value of a PET measure from striatum, a subcortical structure in which Aβ is typically detected at autopsy only after cortical deposition [10] and in which corresponding PET measures are readily available [3]. We reasoned that because striatal involvement reflects a more progressive amyloidosis at postmortem, an *in vivo* striatal PET measure could provide predictive information that differs from the typical cortical PET measure and have a stronger relation to cognitive decline. Specifically, we used data from two large observational studies to test two hypotheses: First, we used serial PET imaging data to confirm *in vivo* that Aβ accumulates later in striatum than in cortex. Second, we hypothesized that participants with elevated cortical Aβ plus elevated striatal Aβ had a more advanced clinical syndrome, greater tau deposition, lower hippocampal volume, and greater cognitive decline than those with elevated cortical and low striatal Aβ.

## 2. Methods

### 2.1. Study design

This prospective study analyzed data from 1433 participants enrolled in either the Harvard Aging Brain study (HABS) or the Alzheimer’s Disease Neuroimaging Initiative (ADNI). HABS is an ongoing, longitudinal, monocentric study conducted at Massachusetts General Hospital (USA). ADNI is an ongoing, longitudinal, multicenter study conducted in 59 sites across the USA and Canada. Eligibility criteria and study designs of HABS and ADNI are similar: Normal participants, aged 55 to 94 years, are recruited from the community together with patients having mild cognitive impairment (MCI) or Alzheimer’s dementia (AD). MRI and PET imaging data are acquired shortly after inclusion (baseline); cognitive follow-up data are acquired annually for virtually all participants, and imaging follow-up data are available in a subset of the participants. Exclusion criteria include history of alcoholism, drug abuse, head trauma, or serious medical or psychiatric condition. Cholinesterase inhibitors and memantine are only allowed in MCI or AD patients if stable for three months before screen. Antidepressants are allowed for both normal and impaired participants if they are not depressed at the time of screen and do not have a history of major depression within the past 1 year. In both cohorts, institutional review board approvals and informed consents were obtained before all procedures.

For the purpose of this research, we did not include participants who had no PET data available. We also excluded from our analyses cognitive data that were acquired more than six months before PET was conducted. PET-Aβ data used in this study were first acquired in ADNI in May 2010 and in HABS in September 2010. Data used in the present report were thus collected between November 2009 and July 2016, when data from both cohorts were downloaded for analyses. The clinical dementia rating (CDR) obtained at the clinical assessment closest to baseline PET was used as a diagnostic criterion for CN (CDR = 0), MCI (CDR = 0.5), and AD dementia (CDR ≥ 1). Baseline MMSE in CN and MCI participants was greater than or equal to 24/30.

### 2.2. Neuropsychology

Cognitive performances, as assessed using the Mini Mental State Examination (MMSE) score, episodic memory, and executive function tests were evaluated at baseline and then followed annually in both HABS and ADNI. Z-scores specific to each cohort were computed: the Preclinical Alzheimer Cognitive Composite (PACC) [13] in HABS and memory [14] and executive function [15] factor scores in ADNI. Cognitive follow-up data were not analyzed in participants who had AD dementia at baseline because our aim in analyzing cognitive changes was to evaluate the predictive power of striatal compared to cortical PET-Aβ in nondemented individuals. Patients with AD dementia at baseline were only included in the descriptive statistics and in the longitudinal PET data analyses.

### 2.3. Imaging

Aβ burden was assessed using C^11^ Pittsburgh compound B (PiB) in HABS and F^18^ Florbetapir (FBP) in ADNI. PiB data were expressed as distribution volume ratios (DVR; 40–60 minutes) scaled on cerebellar gray after partial volume correction using geometric transfer matrix [16]. FBP data were expressed as standard uptake volume ratios (SUVs; 50–70 minutes) scaled on a composite reference region including whole cerebellum and hemispheric white matter [17]. Data from both cohorts were coregistered to each participant’s MRI and anatomically parcellated using FreeSurfer v5.1. We used previously published cortical aggregates [16,17], specific to each cohort and tracer, and the striatum region consisted of a volume-weighted average of putamen and caudate FreeSurfer regions for both cohorts.

Hippocampal volume was obtained from the MRI closest to baseline PET using FreeSurfer and residualized for intracranial volume. Tau deposits were evaluated in the inferior temporal gyrus using F^18^ Flortaucipir-PET (aka T807 or AV1451) in a subset of HABS participants (n = 187). Flortaucipir-PET data were acquired 80 to 100 minutes after injection and expressed as SUVr, using cerebellar gray as reference region and partial volume correction [16]. Because tau PET was only available in HABS from the middle of the study (from July 2013 onwards), we did not relate tau-PET to the baseline PiB-PET session but to the closest PiB-PET session from tau PET, with a median lag time between PET of one month (0.0–11.1). Because tau-PET data were only available in a small number of impaired participants, tau-PET data from MCI participants (n = 41) were analyzed together with data from participants with AD (n = 5). Excluding these five participants decreased the size of the effect but did not modify otherwise the observations.

### 2.4. PET-Aβ cut-points

Cortical PET-Aβ thresholds for each tracer were defined on the basis of published literature [16–18]. Striatal thresholds were defined on the basis of autopsy data indicating that fibrillar Aβ in striatum is very rare unless it is also present in cortex [10–12], that is, striatal PET signal would not be expected in CN individuals who were below the PET threshold for elevated cortical Aβ. Thus, we defined the threshold for elevated striatal Aβ as being greater than the 99th percentile of the striatal binding in those CN whose cortical binding was below published threshold. Although both PiB and FBP distributions in cortical and striatal regions were significantly different from the normal distribution (Lilliefors tests, all *P* < .001), cortical and striatal bindings among those with low Aβ were normally distributed (Lilliefors, all *P* > .50). On the basis of these thresholds, we posited three PET stages of cerebral Aβ:

1. PET stage 0: low cortical, low striatal PET signal
2. PET stage 1: high cortical, low striatal PET signal
3. PET stage 2: high cortical, high striatal PET signal

### 2.5. Statistics

We first tested the hypothesis that PET detects Aβ first in cortex (stage 1) and then in striatum (stage 2), using longitudinal PET data. We reported for each PET-Aβ stage the proportion of individuals who transitioned to another stage after the 3-year follow-up. An exact binomial test compared the different possible outcomes for an individual who had stage 0 at baseline, inquiring whether it was more likely to cross the striatal or the cortical threshold. Then, we used a Fisher’s exact test comparing the proportion of individuals in stage 1 who transitioned to stage 2 to the proportion of individuals in stage 0 who transitioned to stage 2 to confirm a temporal progression from stage 0 to stage 1 and then from stage 1 to stage 2. Finally, using an exact binomial test, we verified that forward transitions from stage 1 to stage 2 were more frequent than backward transitions from stage 1 to stage 0.

We next compared the distributions of PET-Aβ stages across diagnostic groups (CN, MCI, and AD) to determine whether high striatal Aβ would be associated with clinical progression. Within the diagnostic groups, we used linear regressions to assess the cross-sectional association between PET-Aβ stage and tau-PET signal, hippocampal volume, and cognition. We fit linear mixed-effect models with a random intercept per subject to evaluate the longitudinal association between PET-Aβ stage and subsequent cognitive decline. We directly compared cortical and striatal PET-Aβ measures by entering them simultaneously in linear mixed-effect models predicting cognition over time. This was performed with continuous PET measures predicting different cognitive outcomes in both cohorts separately and with three-category staging schemes (a cortical only and a cortico-striatal) predicting MMSE in both cohorts merged. We used the slope of decline and residual variance obtained in linear mixed models to conduct power estimations [19] for hypothetical clinical trials using different outcomes. All models were adjusted for age, sex, and education (as well as cohort and clinical diagnoses when applicable). We report two-tailed *P* values (α = 0.05). The statistical toolbox of MATLAB v9.0.1 (R2016a) was used for all statistics.

## 3. Results

### 3.1. Cohort characteristics

Table 1 shows the characteristics of the participants included in the study and highlights the differences between diagnostic groups within HABS and ADNI. We observed very few differences between cohorts: CN participants in HABS were less educated (0.7 year, [confidence interval 95%, 0.3–1.2], two-sample t-test = 3.4, *P* < .001) than those in ADNI, and demented participants in HABS were younger (7.2 year [3.6–11.8], t = 3.7, *P* < .001) and had lower MMSE (2.7 points [1.0–4.4], t = 3.2, *P* = .002) than those in ADNI. Demographics and MMSE were not different between MCI participants from both cohorts.

PET-Aβ stage classification by diagnostic group is also given in Table 1. Stage 2 was more frequently observed in HABS than in ADNI (CN: X^2^ = 6.0, *P* = .014; MCI: X^2^ = 3.1, *P* = .080; AD: X^2^ = 4.7, *P* = .030). Only 2 of 787 (0.25%) impaired participants (who did not serve for defining the thresholds) had high striatal Aβ and low cortical Aβ; therefore, these two data points were excluded, and only three stages were explored.

### 3.2. Longitudinal changes in PET stages indicate a sequence from cortex to striatum

**Fig. 1.**
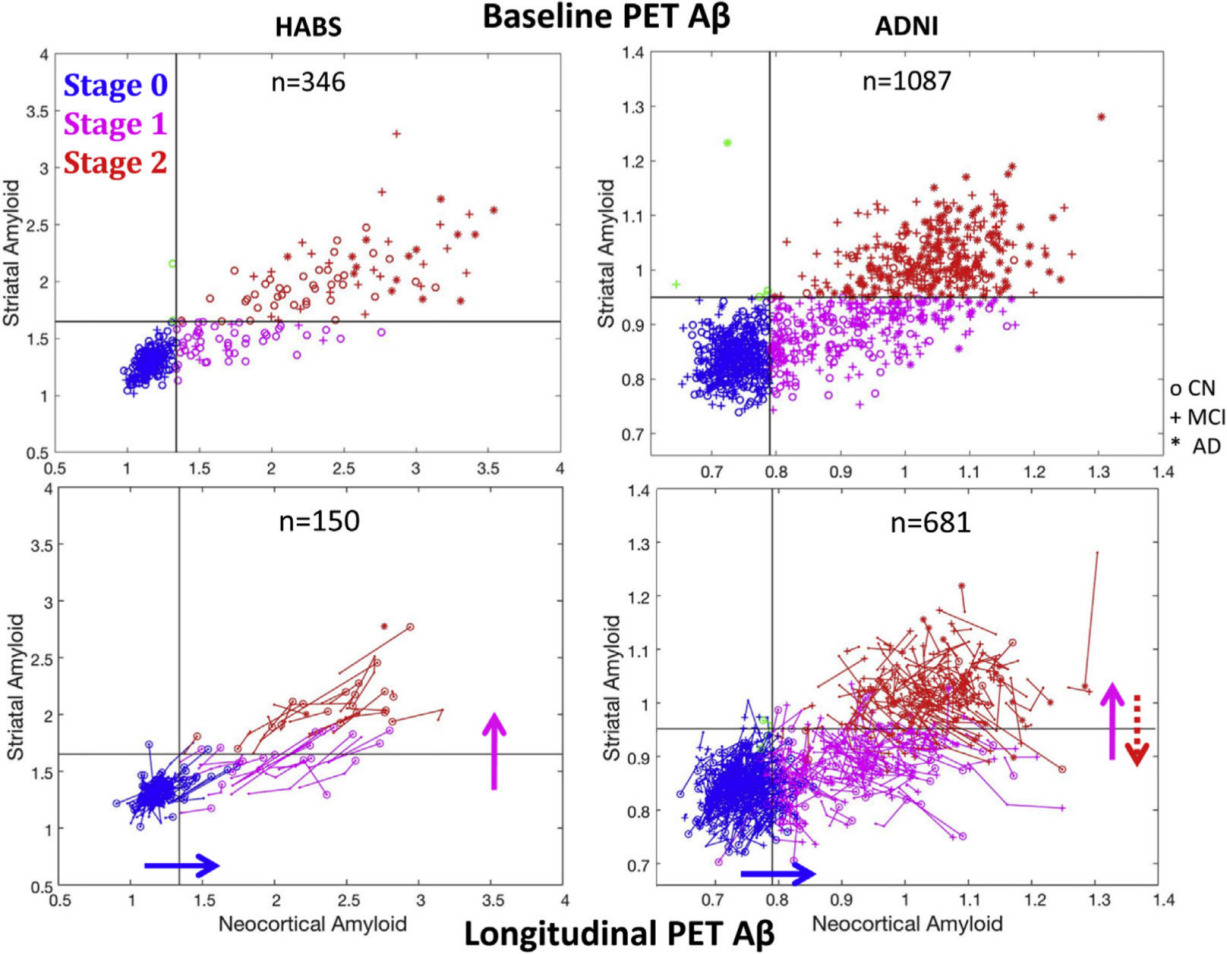
Baseline and longitudinal PET data support a classification in three sequential stages according to cortical and striatal signals. Top row: PET data in the striatum versus cortex from the HABS (C^11^PiB) and the ADNI (F^18^Florbetapir). Striatum can distinguish two groups among individuals with high cortical Aβ. Subjects with PET-Aβ stage 1 have striatal signal in the same range compared with low-Aβ CN participants, and subjects with PET-Aβ stage 2 participants have striatal signal above the 99^th^ percentile of low-Aβ normal participants. Bottom row: Spaghetti plots showing longitudinal change in striatal and cortical Aβ PET in both cohorts over a 3-year follow-up (Table 2 and text for statistics). Plain arrows indicate the most frequent transition observed. The dotted red arrow highlights the backward transitions observed in ADNI only. Abbreviations: Aβ, amyloid β; ADNI, Alzheimer’s Disease Neuroimaging Initiative; CN, clinically normal; HABS, Harvard Aging Brain study.

Cross-sectional data indicated that among those with high cortical Aβ, striatal Aβ was variable compared to the normal range (Fig. 1, top row) possibly because it increases later compared with cortical Aβ, as we hypothesized. We tested whether serial PET measures showed a sequence identified by the three stages using 3-year follow-up PET data from 829 participants (Fig. 1, bottom). Table 2 gives the sample frequencies by baseline and follow-up PET-Aβ staging in each cohort. Most participants did not change stage over the study. As expected, the most frequent transition for participants in PET-Aβ stage 0 at baseline was to PET-Aβ stage 1 (observed in 11.1%); the most frequent transition for individuals in PET-Aβ stage 1 at baseline was to PET-Aβ stage 2 (12.7%). Less than 1% of participants in PET-Aβ stage 0 transitioned directly to stage 2, indicating that a participant with low Aβ in both regions at baseline was more likely to cross the cortical threshold than the striatal threshold during the follow-up (exact binomial test: *P* < .001). A Fisher’s exact test demonstrated that the probability of transitioning to stage 2 was statistically higher for participants in stage 1 at baseline than for participants in stage 0 (*P* < .001), indicating that the elevation in striatal Aβ is preceded by an elevation in cortical Aβ. Backward transitions (PET-Aβ stage 2 → 1 → 0) were only observed in ADNI. Subjects in stage 1 were nevertheless more likely to move forward to stage 2 than backward to stage 0 (exact binomial test: *P* = .011). To provide the reliability of cortical and striatal measures for both tracers, we calculated their coefficient of variation (COV = standard deviation of change/mean change) and showed that striatal PiB (COV = 1.78) had similar variability than cortical PiB (COV = 1.46); in contrast, striatal FBP (COV = 8.41) was much more variable than cortical FBP (COV = 2.30).

### 3.3. High striatal Aβ corresponds to disease progression

**Fig. 2.**
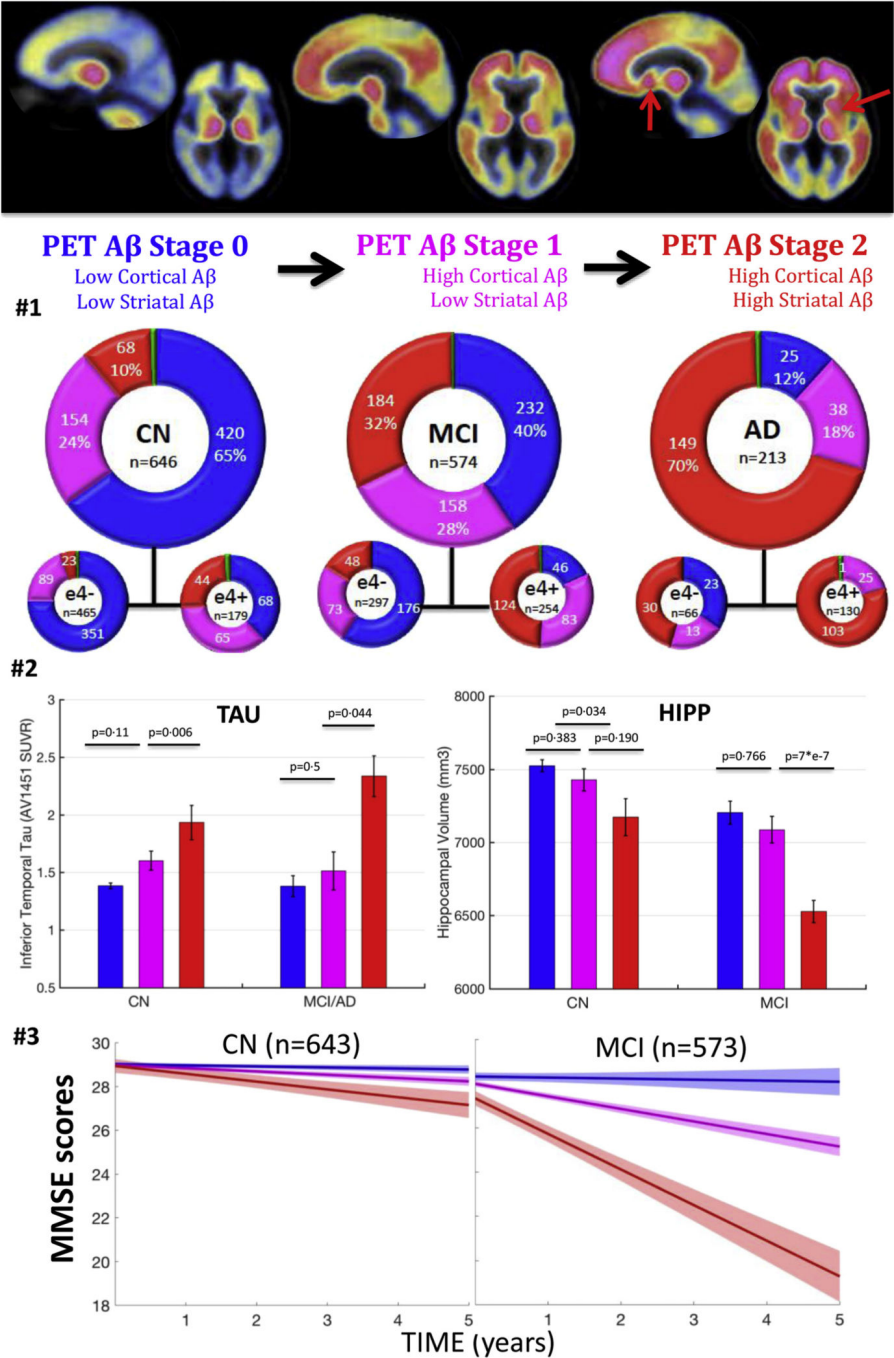
Clinical impairment and tau-PET signal increase, whereas hippocampal volumes decrease with PET-Aβ stages. Top row: illustration of the proposed staging system—PiB-PET images exemplifying the three PET stages 0, 1, and 2 (mean image across HABS participants in each stage). The red arrows indicate striatum showing high PET signal in some but not all individuals with high cortical Aβ. Second row: number of participants in each PET-Aβ stage split by clinical diagnostic groups. The blue color indicates individuals with low cortical, low striatal Aβ (stage 0). The green color indicates individuals with high striatal, low cortical Aβ (,1% of participants). The pink color indicates individuals with high cortical, low striatal Aβ (stage 1). The red color indicates individuals with high cortical and high striatal Aβ. Third row: tau-PET signal and adjusted hippocampal volume as a function of PET-Aβ stages. Raw data plot (bars are standard errors); *P* values are adjusted for demographics and cohort. Last row: longitudinal MMSE by baseline PET-Aβ stages, adjusted for demographics and cohort. PET-Aβ stage 2 individuals have the fastest cognitive decline than any other group. Error bars are 95% confidence intervals. See first two rows of Table 3 for statistics. Abbreviations: Aβ, amyloid β; AD, Alzheimer’s dementia; CN, clinically normal participants; HABS, Harvard Aging Brain study; MCI, mild cognitive impairment.

To inquire whether PET-Aβ stages were associated with clinical syndrome, we assessed the proportions of CN, MCI, and AD participants in each PET-Aβ stage (Fig. 2, second row). Relative proportions of participants within each PET-Aβ stage (0, 1, and 2) differed across diagnostic groups. In particular, the proportion of participants in stage 2 increased from CN to MCI to AD group, whereas the proportion in stage 0 decreased. Across diagnostic groups, stage 1 is more consistent (18%–28%) reflecting that it is transitional, and the proportional representation by apolipoprotein E status was in the expected direction; there were no participants in stage 0 among ε4 carriers with AD.

PET-Aβ stages were consistently reflected in the subset of participants with available tau-PET data (Fig. 2, row 3, left). Impaired participants in PET-Aβ stage 2 had significantly higher tau-PET signal than those in stage 1 (0.79 ± 0.38 SUVr [estimate ± standard error of the fit], *P* = .044); by contrast, impaired participants in stage 1 and in stage 0 had similar levels of tau-PET signal (0.29 ± 0.41 SUVr, *P* = .5) although the small sample size calls for cautious interpretation. Similar results were observed in CN group, with higher tau-PET signal in stage 2 than in stage 1 (0.36 ± 0.13 SUVr, *P* = .006). By contrast, stage 1 CN participants had a marginal, nonsignificant increase in tau-PET signal compared with stage 0 ones (0.18 ± 0.11 SUVr, *P* = .11).

PET-Aβ stages related to hippocampal volumes (Fig. 2, row 3, right). MCI participants in stage 2 had smaller hippocampi compared with MCI participants in stage 0 (−583 ± 100 mm^3^, *P* < .0001) or in stage 1 (−551 ± 110 mm^3^, *P* < .0001). CN participants in stage 2 also had lower volumes than CN participants in stage 0(−227 ± 106 mm^3^, P5.032), but the comparison between stages 2 and 1 did not reach statistical significance (−159 ± 120 mm^3^, *P* = .19). MCI (−31 ± 106 mm^3^, *P* = .77) and CN (−68 ± 78 mm^3^, *P* = .38) participants in stage 1 only had a marginal, nonsignificant volume difference compared with their stage 0 counterparts. Unlike hippocampus, striatal volumes did not systematically differ by diagnoses or PET-Aβ stages.

Consistent with tau-PET and MRI observations, PET-Aβ stages related to concurrent MMSE performances in MCI group (Fig. 2, row 4 at baseline = time 0). MCI participants in PET-Aβ stage 2 (baseline MMSE: 27.4 ± 1.8) had lower performances than MCI participants in stage 1 (28.0 ± 1.7, *P* < .0001), who had slightly lower performances than MCI participants in stage 0 (28.3 ± 1.5, *P* = .07). By contrast, baseline MMSE did not differ across PET-Aβ stages in CN group (stage 0: 29.1 ± 1.2, stage 1: 28.9 ± 1.1, stage 2: 28.9 ± 1.0; *P* > .17). This was also the case for PACC (*P* > .15) and the ADNI-factor scores (*P* > .11). Normal older individuals with high striatal Aβ had thus imaging markers suggestive of Alzheimer’s disease progression but no cognitive impairment at the time of PET.

### 3.4. High striatal Aβ predicts faster cognitive decline than high cortical Aβ

Linear mixed models demonstrated that MCI in stage 2 had faster subsequent decline in MMSE (−1.16 ± 0.06 points/y) than MCI in stage 1 (−0.25 ± 0.06 points/y) and stage 0 (−0.07 ± 0.08 points/y; Fig. 2, last row). Similar results were observed predicting ADNI-factor scores. Stage 2 CN also had the fastest cognitive decline, regardless of the outcome analyzed (Table 3, rows 1–7).

We then aimed to confirm the regional specificity of striatal Aβ and control for the cortical Aβ burden within high-Aβ individuals, to test whether the striatal measure was merely serving as a proxy for a very high cortical measure or explains additional unique variance. To this end, we predicted cognitive decline in high-Aβ CN participants with continuous PET-Aβ measures in both cortex and striatum. Cognition was better predicted by Aβ in striatum than Aβ in cortex (HABS-PACC: −0.22 ± 0.11 z-scores/y per striatal PiB-DVR, *P* = .05, vs. 0.06 ± 0.08 z-scores/y per cortical PiB-DVR, *P* = .47; average ADNI-factor score: −0.72 ± 0.35 z-scores/y per striatal FBP-SUVr, *P* = .04, vs. 0.04 ± 0.27 z-scores/y per cortical FBP-SUVr, *P* = .88). Post hoc tests showed that the slope of decline of cortex and striatum significantly differ from each other (HABS: *P* = .001, ADNI: *P* = .092). Similar results were obtained for MMSE in both cohorts; striatal PET-Aβ had closer association with cognitive decline than cortical PET-Aβ.

Because striatal and cortical Aβ are correlated (R^2^ between striatal and cortical within-high Aβ CN: PiB = 0.50 and FBP 5.39), disentangling their contributions in overlapping staging schemes requires large samples. To compare staging with and without striatal information, we thus sought to merge cohorts and predicted MMSE decline in 1216 nondemented individuals. We compared our three-category staging system including striatal Aβ (−0.28 ± 0.05 points/y per stage, *P* < .0001) to a three-category staging system that only included cortical Aβ (−0.08 ± 0.05 points/y per stage, *P* = .10) and demonstrated the superiority of a staging system including striatum. The three cortical categories were low, moderately high, and very high cortical Aβ. The median cortical value of high-Aβ individuals distinguished moderately high from very high cortical Aβ. Combining staging systems allowed disentangling striatal and cortical Aβ in a 2-by-2 design: stage 1+ (moderately high cortex, low striatum), stage 1++ (very high cortex, low striatum), stage 2+ (moderately high cortex, high striatum), and stage 2++ (very high cortex, high striatum).

**Fig. 3.**
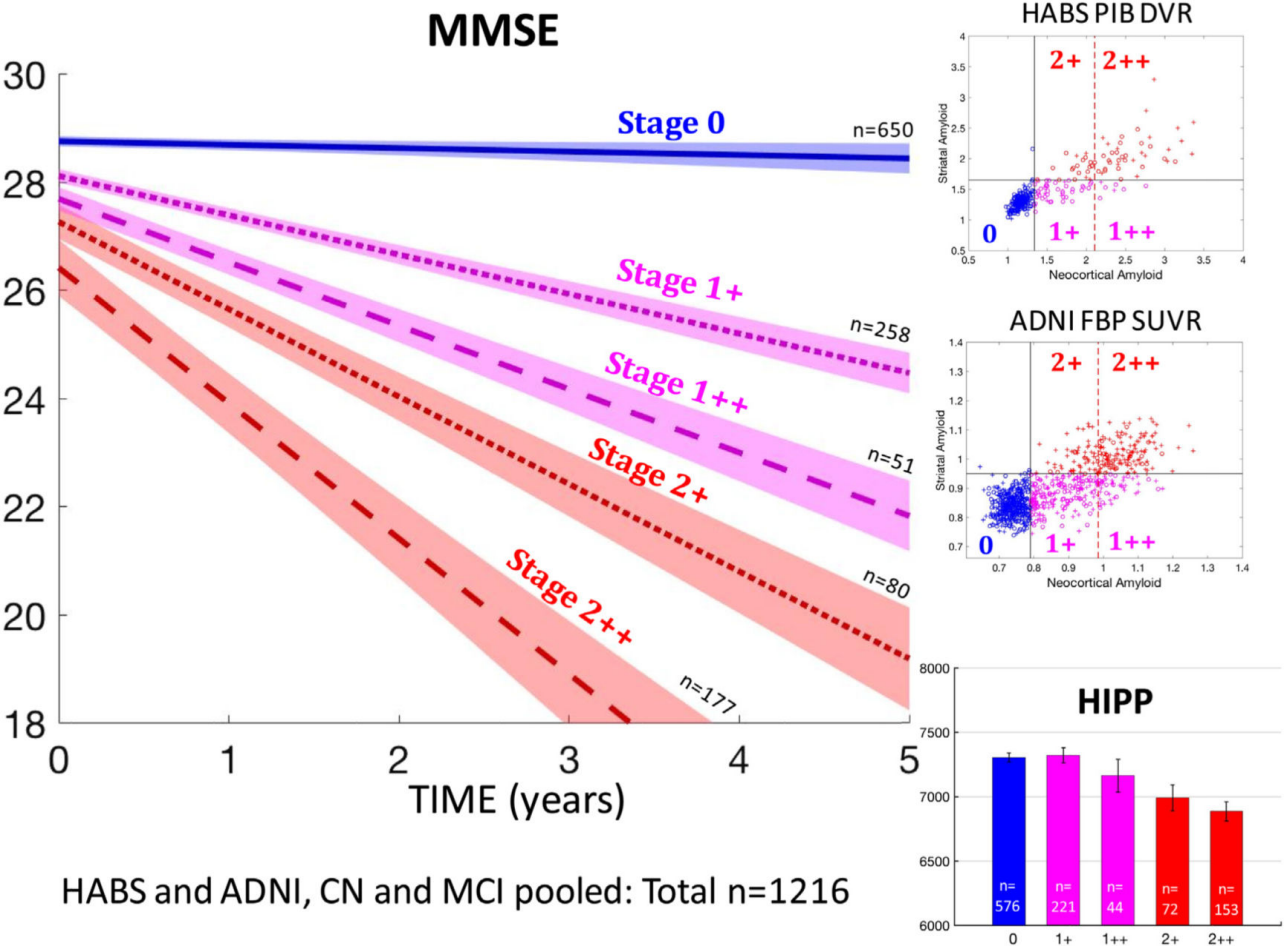
MMSE decline and hippocampal atrophy are more severe in individuals with high striatal Aβ than in individuals with very high cortical Aβ. Top and middle rows on the right: In both HABS and ADNI, individuals with high cortical Aβ are subdivided into four PET-Aβ substages, using both a striatal and a very high cortical Aβ threshold—1+: moderately high cortex, low striatum; 1++: very high cortex, low striatum; 21: moderately high cortex, high striatum; 2++: very high cortex, high striatum. Bottom right: hippocampal volumes by PET-Aβ substages in nondemented older adults. Striatal PET-Aβ, but not very high cortical PET-Aβ, is associated with lower hippocampal volumes. Left: MMSE decline by PET-Aβ substages in nondemented older adults. Groups with high striatal Aβ (2+ and 2++) demonstrated the fastest decline. Error bars are 95% confidence intervals. See the last row of Table 3 for statistics. Abbreviations: Aβ, amyloid β; ADNI, Alzheimer’s Disease Neuroimaging Initiative; HABS, Harvard Aging Brain study.

We observed that high striatal Aβ individuals (stages 2+ and 2++) had faster MMSE decline than low striatal Aβ individuals (stages 1+ and 1++), after accounting for cortical Aβ stage (Fig. 3). Specifically, participants in stage 2+ had faster decline than participants in stage 1++ (−0.22 ± 0.09 points/y, *P* = .022), although cortical Aβ burden was higher in stage 1++ than that in stage 2+ (Table 3, last row).

High striatal Aβ was associated with smaller hippocampi among individuals with moderately high Aβ (Fig. 3, bottom right; stage 2+ vs. 1+, −359 ± 117 mm^3^; *P* = .002) and very high cortical Aβ (stage 2++ vs. 1++, −289 ± 145 mm^3^; *P* = .047). By contrast, very high cortical Aβ was not associated with significantly lower volumes than moderately high cortical Aβ (stage 2++ vs. 2+, −113 ± 121 mm^3^, *P* = .35; stage 1++ vs. 1+, −183 ± 141 mm^3^, *P* = .20). Thus, striatal Aβ does not merely reflect the global Aβ burden but indicates a more severe pathology, with greater cognitive decline and atrophy. Because striatal Aβ has closer association with cognitive decline than cortical Aβ, including striatal Aβ in a staging system increases its predictive value.

### 3.5. Implications for clinical trials and dementia prediction

We estimated the number of participants a clinical trial would need to enroll to detect a slowing of cognitive decline of 30% (2 arms over 4-year duration with annual assessments; 80% power; a5 0.05) using an inclusion criteria elevation in cortical Aβ (all high-Aβ individuals) or elevation in striatal Aβ (PET-Aβ stage 2). To predict PACC in CN, a trial would need to enroll 440 high-Aβ individuals per arm compared with the 294 needed for stage 2 (33% sample size reduction; Supplementary Fig. S1). To predict ADNI memory factor in CN, 607 high-Aβ or 215 stage-2 individuals are required per arm (65% reduction). To predict ADNI executive function factor in CN, 479 high-Aβ or 122 stage-2 individuals (75% reduction) are required. To predict MMSE, 899 high-Aβ or 411 stage-2 individuals are required in CN (54% reduction) and 193 high-Aβ or 78 stage-2 individuals in MCI (60% reduction). Thus, using different samples and outcomes, sample sizes were consistently reduced (by 33%– 75%) when using striatal instead of cortical Aβ as eligibility criteria. Screen failure rates would however increase by requiring high striatal Aβ at inclusion.

We finally computed survival curves, predicting whether nondemented participants would progress to dementia during the follow-up (Supplementary Fig. S2) using MMSE cutoffs to characterize dementia as mild (MMSE < 24), moderate (MMSE < 20), or severe (MMSE < 12). After a median follow-up of 3 years, 3% of individuals with stage 0 at baseline had mild dementia, but none had moderate or severe dementia. Eleven percent of individuals with stage 1 at baseline were demented, including 8% with mild and 3% with moderate dementia, but none had severe dementia. Forty-two percent of individuals with stage 2 at baseline were demented, including 21% with mild dementia, 15% with moderate dementia, and 6% with severe dementia.

## 4. Discussion

We assessed a novel method of measuring brain Aβ burden, which defined three PET stages of amyloidosis: low Aβ, high Aβ in cortex only, and high Aβ in both cortex and striatum. In two large observational studies, we classified 1433 participants and confirmed with serial PET measures that individual trajectories advance successively through the three stages and from normal to MCI to AD. We found the three stages to be related to levels of tau burden, hippocampal atrophy, and progression to greater cognitive impairment and dementia. We found that elevated striatal Aβ was not merely a proxy for very high levels of cortical Aβ. Our striatal measure was able to predict cognitive decline better than cortical Aβ, using continuous data or categories.

PET-Aβ was a breakthrough technology for detecting *in vivo* Alzheimer’s pathology. Cortical deposition, however, becomes detectable long before cognitive impairment. Transition rates between biomarker states estimated that 5 to 10 years separate the detection of cortical Aβ from the detection of downstream phenomena such as atrophy [20]. An in-press study observed that 32.2% of high-Aβ CN individuals progress to MCI within the next 4 years, whereas 88.2% would progress after 10 years [21]. However, most clinical trials have much shorter durations than a decade. The present study suggests that using striatum to identify older adults at risk for cognitive impairment would result in shorter times to progression. Future studies will investigate the risk of progression to AD dementia at the individual level in MCI patients with and without striatal amyloid, but current evidence suggests a lower risk for AD in MCI patients with low striatal amyloid and a higher risk for non-AD pathologies as an alternative cause of their cognitive impairment. Evaluating striatal Aβ may be of particular relevance in prevention trials using PET-Aβ as eligibility criteria [22]. We demonstrated that using striatum would give additional power in such trials but at the cost of additional screen failures. Cost benefits of using cortex or striatum as eligibility criteria will depend on the specific aims of the trial, targeting earlier or later preclinical Alzheimer’s stages. Some clinical trials may consider using a striatal PET threshold as inclusion criteria to ensure a more rapid progression of the participants. Other clinical trials may exclude individuals with striatal Aβ to enroll exclusively participants with incipient amyloidosis. Recent development in biomarker technology suggests that it will soon be possible to detect Aβ positivity in plasma [23,24]. This advance will allow screening Aβ pathology in the population at large, but confirmation and refinements using image-based regional staging will certainly be valuable to increase specificity.

This study also provides longitudinal evidence that striatal Aβ occurs later in the disease course than cortical Aβ, consistent with the hypothesis formulated from autopsy data [10] and with the observation of additional clinical-pathological features associated with striatal Aβ. In contrast, two familial Alzheimer’s studies showed early striatal PET-Aβ signal [25,26]. Although longer follow-up periods are required, these observations suggest that genetic factors might influence regional Aβ spread. Consistent with a genetic hypothesis, early striatal Aβ was observed in Down syndrome [27]; and among high Aβ individuals, we observed more ε4 carriers with high striatal Aβ than with ε4 noncarriers, suggesting that e4 not only increases the global Aβ load but also modifies its regional distribution.

### 4.1. Limitations and future directions

We noted that high striatal PET-Aβ was less frequently observed in ADNI than in HABS (Table 1) and occasionally inconsistent after follow-up (Table 2), with higher variability in the change measure for FBP than for PiB. These observations likely reflect FBP binding to white matter around and within striatum. Although they indicate a lower confidence in FBP than in PiB classification [28], cognitive results were highly similar across cohorts. Most ADNI participants with dementia had elevated striatal FBP (61%), and the few CN (8%) or MCI (31%) participants with high striatal FBP had the fastest cognitive decline. Altogether, these findings suggest that FBP is not as sensitive as PiB to detect striatal pathology, but both FBP and PiB striatal signals seem specific to decliners. Consistent with the view that FBP is able to detect striatal Aβ, a recent ADNI study showed that striatal FBP signal correlated with Aβ CSF measures [29].

We did not measure Aβ further along Thal Phases because brainstem and cerebellum are often used as reference regions in PET studies and are less likely to be affected in CN. We also did not assess allocortical Aβ because Thal Phase 2 appears difficult to identify using PET [30,31].

Mechanistically, whether the expansion of amyloid into striatum leads directly to cognitive decline, indicates the duration or severity of Aβ burden, or signals an increase in tau pathology remains to be determined. The small sample with available tau-PET data did not allow us to fully disentangle the contributions of cortical and striatal Aβ to tau pathology. Only 67 individuals with tau-PET data had elevated cortical Aβ, preventing us from evaluating the substaging system or to make both PET measures competing in the same model. Postmortem data indicate a stronger association of Braak staging with striatal than cortical Aβ [12], and a study on Parkinson’s disease with dementia showed that in addition to Lewy bodies, the presence of striatalAβ was specific for AD pathology, including Braak stage III to VI, whereas cortical Aβ plaques were sensitive but not as specific [32].

Recent work suggests strong correlations between tau-PET signal and cognitive performances [33–35], whereas we only showed marginal, nonsignificant correlations between high striatal Aβ and cross-sectional cognition. This observation suggests an indirect effect of striatal Aβ on cognition, which may be partially tau mediated.

The present study did not address the question of a potential striatal dysfunction induced by Aβ pathology, nor whether some cognitive domains would be more specifically impaired. Previous work in familial cases suggested a strong relation between striatal Aβ and executive functions [36]. So far, no studies on sporadic Alzheimer’s have focused on functional imaging of striatum in relation to striatal Aβ pathology. Additional work is also needed to distinguish the respective contributions of striatal Aβ to the onset of neuropsychiatric symptoms. Finally, the use of striatal Aβ as a potential biomarker outcome in anti-Aβ clinical trials should be investigated.

## Supplementary Material

1

2

## Figures and Tables

**Table 1:** Characteristics of the participants

	Harvard aging brain study			Alzheimer’s disease neuroimaging initiative		
Clinical diagnoses	CN (n = 279)	MCI (n = 51)	AD (n = 16)	CN (n = 367)	MCI (n = 523)	AD (n = 197)
Age, y	73.7 (6.1)	72.8 (8.8)	68.1 (9.7)^†^	74.7 (6.6)	72.7 (7.9)^†^	75.3 (7.8)
Education, y	15.8 (3.0)	16.5 (3.1)	15.5 (2.3)	16.5 (2.6)	16.1 (2.7)*	15.9 (2.7)*
Female, %	59.5%	35.3%*	31.3%*	53.1%	43.2%*	41.6%*
E4 carriers, %	29.0%, missing = 3	40.6%,^†^ missing = 19	75.0%,^†^ missing = 12	27.8%, missing = 0	46.7%,^†^ missing = 0	64.5%,^†^ missing = 0
Baseline MMSE	29.0 (1.0)	27.6 (1.4)^†^	20.1 (4.1)^†^	29.0 (1.2)	28.0 (1.7)^†^	22.8 (3.1)^†^
PET-Aβ stages, 0/1/2; N and %	192/44/40; 70/16/14%	23/6/22^†^; 45/12/43%	1/0/15^†^; 6/0/94%	228/110/28; 62/30/8%	209/152/162^†^; 40/29/31%	24/38/134^†^ 12/19/68%
Serial PET interval, y	3.4 (1.3), missing = 135	2.3 (0.9), missing = 47	3.2 (1.5), missing = 14	2.4 (0.8), missing = 99	2.6 (0.9),* missing = 161	2.2 (0.6), missing = 146
Cognitive follow-up, y	3.6 (1.3)	2.7 (2.2) ^†^	N/A	3.1 (1.4)	3.3 (1.4)*	N/A

Abbreviations: Aβ, amyloid β; AD, Alzheimer’s dementia; CN, clinically normal participants; MCI, mild cognitive impairment.

NOTE. * and ^†^ represent values significantly different from CN of the corresponding cohort.

* *P* < .050

† *P* < .001

NOTE. Statistics: two-sample t-tests except for sex, genotype, and PET-Aβ stages for which χ^2^ are used.

NOTE. Age and MMSE are given at the closest time of baseline PET.

NOTE. N/A represents that cognitive follow-up data were not analyzed in participants who had AD dementia at baseline.

**Table 2:** PET-Aβ stage frequencies after a 3-year PET follow-up

	Follow-up PET			
Baseline PET stage	PET stage	HABS	ADNI	Total
PET-Aβ stage 0	PET-Aβ stage 0	N = 91, 85.0%	N = 293, 88.3%	N = 384, 87.5%
Low cortical	PET-Aβ stage 1	N = 14, 13.1%	N = 34, 10.5%	N = 49, 11.1%
Low striatal	PET-Aβ stage 2	N = 1, 0.9%	N = 3, 0.9%	N = 4, 0.9%
HABS, N = 107	Low cortical	N = 1, 0.9%	N = 1, 0.3%	N = 2, 0.5%
ADNI, N = 332	High striatal			
PET-Aβ stage 1	PET-Aβ stage 1	N = 12, 57.1%	N = 157, 85.3%	N = 169, 82.4%
High cortical	PET-Aβ stage 2	N = 9, 42.9%	N = 26, 9.2%	N = 26, 12.7%
Low striatal	PET-Aβ stage 0	N = 0, 0.0%	N = 10, 5.4%*	N = 10, 4.9%
HABS, N = 21	Low cortical	N = 0, 0.0%	N = 0, 0.0%	N = 0, 0.0%
ADNI, N = 184	High striatal			
PET-Aβ stage 2	PET-Aβ stage 2	N = 22, 100.0%	N = 137, 84.0%	N = 159, 85.9%
High cortical	PET-Aβ stage 1	N = 0, 0.0%	N = 26, 16.0%*	N = 26, 14.1%
High striatal	PET-Aβ stage 0	N = 0, 0.0%	N = 0, 0.0%	N = 0, 0.0%
HABS, N = 22	Low cortical	N = 0, 0.0%	N = 0, 0.0%	N = 0, 0.0%
ADNI, N = 163	High striatal			

Abbreviations: Aβ, amyloid β; ADNI, Alzheimer’s Disease Neuroimaging Initiative; HABS, Harvard Aging Brain study.

NOTE. The table shows the number of participants classified in a given PET stage (0, 1, 2) at baseline and at follow-up (total N = 829). Percentages are the proportions of participants in each possible stage at follow-up PET from the total number of participants in a given stage at baseline PET. Results are first split by cohort and then grouped together.

* Indicate backward transitions, which are only observed in ADNI, not in HABS.

**Table 3:** Cognitive decline in and MCI participants with PET-Aβ stages 0, 1, and 2

Sample	Outcome	PET-Aβ stage	Annual Slope	*P* value
			points/y (/30)	
ADNI and HABS MCI	Longitudinal	Stage 0	−0.074	0 vs. 1, *P* = .003
	MMSE	Stage 1	−0.251	1 vs. 2, *P* = e−40
		Stage 2	−1.164	
			points/y (/30)	
ADNI and HABS CN	Longitudinal	Stage 0	−0.037	0 vs. 1, *P* = .002
	MMSE	Stage 1	−0.156	1 vs. 2, *P* = .026
		Stage 2	−0.275	
			z-scores/y	
ADNI MCI	Longitudinal	Stage 0	−0.016	0 vs. 1, *P* = .013
	Memory Factor score	Stage 1	−0.076	1 vs. 2, *P* = e−22
		Stage 2	−0.344	
			z-scores/y	
ADNI CN	Longitudinal	Stage 0	+0.011	0 vs. 1, *P* = .014
	Memory Factor score	Stage 1	−0.074	1 vs. 2, *P* = .267
		Stage 2	−0.141	
			z-scores/y	
ADNI MCI	Longitudinal	Stage 0	+0.037	0 vs. 1, *P* = 6*e−5
	Executive Factor score	Stage 1	−0.053	1 vs. 2, *P* = 6*e−9
		Stage 2	−0.203	
			z-scores/y	
ADNI CN	Longitudinal	Stage 0	−0.013	0 vs. 1, *P* = .293
	Executive Factor score	Stage 1	−0.048	1 vs. 2, *P* = .015
		Stage 2	−0.188	
			z-scores/y	
HABS CN	Longitudinal	Stage 0	+0.053	0 vs. 1, *P* = .001
	PACC	Stage 1	−0.034	1 vs. 2, *P* = .006
		Stage 2	−0.125	
			points/y (/30)	
ADNI and HABS CN and MCI		Stage 0	−0.070	0 vs. 1+, *P* = .027
	Longitudinal	Stage 1+	−0.154	1+ vs. 1++, *P* = .001
	MMSE	Stage 1++	−0.410	1++ vs. 2+, *P* = .022
		Stage 2+	−0.625	2+ vs. 2++, *P* = 2*e−5
		Stage 2++	−0.930	

Abbreviations: Aβ, amyloid β; ADNI, Alzheimer’s Disease Neuroimaging Initiative; CN, clinically normal; HABS, Harvard Aging Brain study; MCI, mild cognitive impairment; PACC, Preclinical Alzheimer Cognitive Composite.

NOTE. Longitudinal cognition by baseline PET-Aβ stages, adjusted for demographics (cohort and clinical diagnosis when applicable). Each box gives the result of a different linear mixed model. For transforming MMSE points in z-scores, divide slope by 1.18 (baseline SD of MMSE in CN). PACC is only available in HABS CN. See text for definition of the different stages. See Figs. 2 and 3 for illustrations using MMSE.

## RESEARCH IN CONTEXT

1. Systematic review: Amyloidosis progression from neocortex to striatum has been supported by multiple postmortem studies. In the 2012 National Institute on Aging–Alzheimer’s Association guidelines for the neuropathologic assessment of Alzheimer’s disease, striatal amyloid was recognized as one of the core criteria (Thal Phase 3). Cognitive decline is associated with cortical amyloid PET, but the association with striatal amyloid PET has not yet been investigated.
2. Interpretation: This study evaluates a pathology-based PET staging of brain amyloidosis using a striatal measure to define a group that is at greater risk than individuals identified using only high cortical amyloid. This group with high striatal amyloid demonstrated greater cerebral tau burden, hippocampal atrophy, and cognitive decline than high-amyloid individuals who had no evidence of striatal pathology.
3. Future directions: Whether the expansion of amyloid into striatum leads directly to cognitive decline signals an increase in tau pathology or simply indicates the duration of amyloid burden requires further exploration.
