# Supplementary figures and images for: PET staging of amyloidosis using striatum
Source: Alzheimers Dement. Author manuscript; Available in PMC 2018 Nov 6. (PMC6219621; doi:10.1016/j.jalz.2018.04.011)

## Slide 1
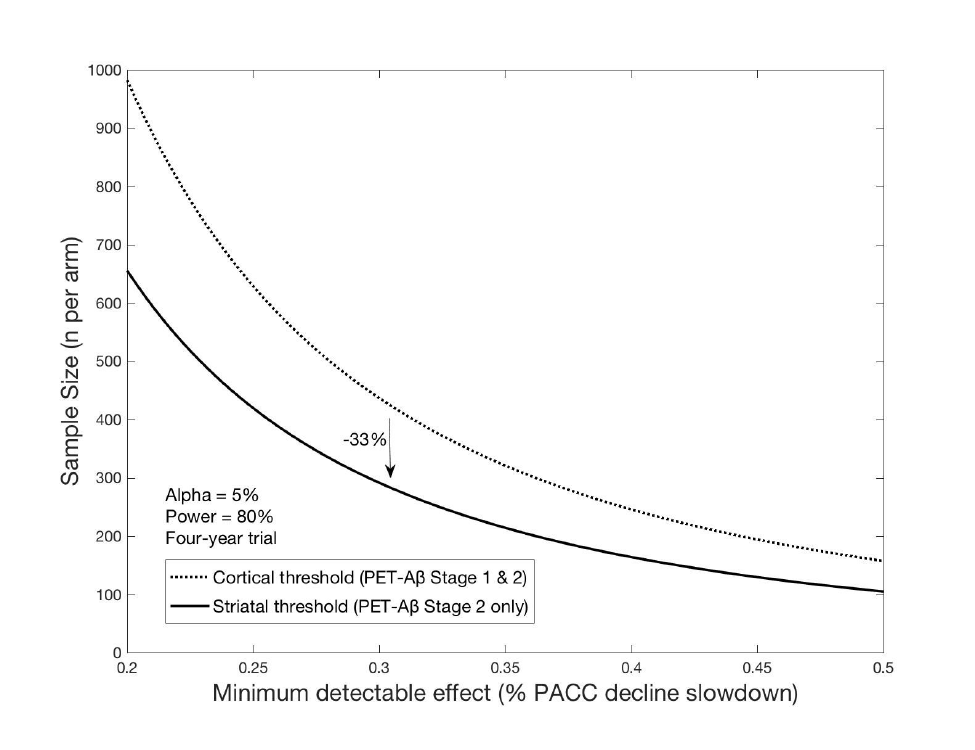

Supplement: 1 [file NIHMS1509490-supplement-1.pptx]
