# Supplementary material for: PET staging of amyloidosis using striatum
Source: Alzheimers Dement. Author manuscript; Available in PMC 2018 Nov 6. (PMC6219621; doi:10.1016/j.jalz.2018.04.011)
Supplement: 2 [file NIHMS1509490-supplement-2.pptx]

## Slide 1
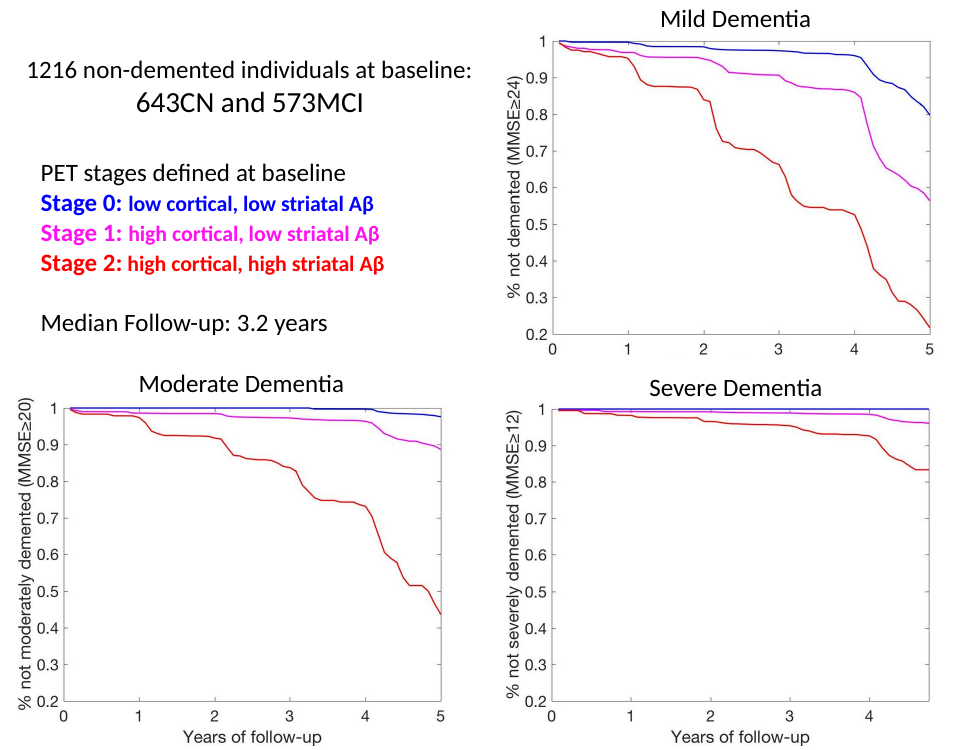

Mild Dementia
1216 non-demented individuals at baseline: 643CN and 573MCI
PET stages defined at baseline
Stage 0: low cortical, low striatal Aβ
Stage 1: high cortical, low striatal Aβ
Stage 2: high cortical, high striatal Aβ
Median Follow-up: 3.2 years
Moderate Dementia
Severe Dementia
